# Development of the “Third-Generation” Hybrid Rice in China

**DOI:** 10.1016/j.gpb.2018.12.001

**Published:** 2018-12-13

**Authors:** Haiyang Wang, Xing Wang Deng

**Affiliations:** 1 School of Life Sciences, State Key Laboratory for Conservation and Utilization of Subtropical Agro-Bioresources, South China Agricultural University, Guangzhou 510642, China; 2 Biotechnology Research Institute, Chinese Academy of Agricultural Sciences, Beijing 100081, China; 3 State Key Laboratory of Protein and Plant Gene Research, Peking-Tsinghua Center for Life Sciences, School of Advanced Agriculture Sciences and School of Life Sciences, Peking University, Beijing 100871, China

## Abstract

**Rice** is a major cereal crop for China. The development of the “three-line” hybrid rice system based on **cytoplasmic male sterility** in the 1970s (first-generation) and the “two-line” hybrid rice system based on photoperiod- and thermo-sensitive genic male-sterile lines (second-generation) in the 1980s has contributed significantly to rice yield increase and food security in China. Here we describe the development and implementation of the “third-generation” hybrid rice breeding system that is based on a transgenic approach to propagate and utilize stable recessive nuclear male sterile lines, and as such, the male sterile line and hybrid rice produced using such a system is non-transgenic. Such a system should overcome the intrinsic problems of the “first-generation” and “second-generation” hybrid rice systems and hold great promise to further boost production of hybrid rice and other crops.

To sustain population growth, crop productivity has increased significantly in the last few decades, in large part, due to the development of new cultivars with increased yields. The identification and utilization of *sd1* (a semi-dwarf mutant) in the 1950s led to the first ‘‘green revolution’’, which helped to solve food shortage in developing countries [1]. Development and adoption of hybrids for several key crops, such as maize and rice, also made a significant contribution to the increased global agricultural productivity. For example, since the development of single-cross hybrid maize varieties in the 1950s, maize yield per unit land area in the United States has increased more than seven folds (from 1287 kg/ha in the 1930s to 9595 kg/ha in 2010) [2].

Hybrid vigor (heterosis) is a universal phenomenon in crops, however, its utilization is still limited to a few crops, which depends on the development of commercially viable hybrid seed production systems [3]. Hybrid seeds, which have evident yield gain and more stable yield potential, are produced by crossing two genetically-distinct inbred parental lines (one male and one female). For monoecious plant, like maize, which has male flower on top of the plant and separated from the female flowers on the same plant, manual or mechanical detasseling has been the predominant method currently to ensure that the female parent only cross-pollinates with the male parent in commercial production of hybrid seeds, despite the sporadic usage of cytoplasmic male sterile (CMS) lines and chemically-induced male sterile lines [4], [5]. However, for monoclinous and autogamous crop, such as rice and wheat, mechanical emasculation of female parent plants is prohibitively expensive and not effective. Thus, alternative female sterility systems are needed.

Rice is one of the major cereal crops for China and worldwide. Asian cultivated rice (*Oryza sativa* L.) can be classified into two morphologically and genetically distinct subspecies, *japonica* and *indica*. Strong hybrid vigor was found in *indica*–*indica* or *indica*–*japonica* hybrids, but not as strong in *japonica*–*japonica* hybrids. To utilize the hybrid vigor in rice, Prof. Longping Yuan has been leading a collaborative effort to identify and develop a commercially viable hybrid system since the 1960s. His team first identified a male-sterile wild rice variety, which carries the gene *wild abortive cytoplasmic male sterility* (*CMS-WA*) [6]. Since then, *CMS-WA* has been introgressed into a variety of rice backgrounds to produce numerous CMS lines, and based on these lines, the “three-line” (male-sterile line, maintainer line, and restorer lines) hybrid breeding system was developed and widely used in China (thus named the “first-generation” hybrid rice). Hybrid rice developed using the ‘‘three-line” hybrid breeding system boosted higher grain yield as compared to inbred varieties [7]. In the 1990s, joint efforts by many Chinese breeders have led to the development of the “two-line” hybrid breeding system, which utilizes photoperiod- and thermo-sensitive genic male-sterile lines (PGMS and TGMS) and male parental lines (thus named the “second-generation” hybrid rice). The first environmentally-sensitive male sterile rice mutant line is discovered by Prof. Mingsong Shi [8]. In commercial production, the fertility of PGMS and TGMS lines is “switched on” for self-propagation and “switched off” for hybrid seed production by changing the locations where the plants are grown. Compared to the “three-line” hybrid system, the “two-line” hybrid system is easier to operate and more efficient in utilization of rice germplasm, and it produces hybrids of higher yields and better grain quality [9]. At their best years after the 1990s, hybrid rice (three-line and two-line hybrids combined) had been planted at ca. 17 million ha in China alone (accounting for about 60% of the total rice cultivation area in China) [7], [10]. However, both the “three-line” and ‘‘two-line” hybrid systems suffer from several intrinsic problems, such as limited germplasm of the restorer lines for the CMS system (only ∼5% of rice germplasm carries the proper nuclear restoration genes for a given CMS gene) and instability of the PGMS and TGMS lines due to vulnerability to uncontrollable weather fluctuations [11], [12].

Recently, with the development of molecular biology and biotechnology, utilization of recessive nuclear genetic male sterility has been proposed as an ultimate approach to engineer a new type of male sterile lines. Recessive nuclear male-sterile lines could provide a stable source of female parent for hybrid seed production (not influenced by environmental changes) and they can be restored by virtually all germplasms that contain the wild type gene for the male sterile mutation. However, it is impossible to propagate the homozygous recessive male sterile line in large quantities for commercial hybrid seed production due to its full male sterility. In 1993, Williams and Leemans demonstrated a strategy to create a transgenic maintainer for a recessive male sterile plant by transforming it with two linked genes, *i.e.* a male fertility restoration gene and a pollen lethality gene [13]. The maintainer line and the male sterile line can be propagated through self-pollination of the maintainer. Later in 2002, similar approaches with an additional third marker gene were proposed by Perez-Prat and van Lookeren Campagne [14]. The male sterile mutant plant transformed with a fertility restoration gene together with a seed color gene and a pollen lethality gene (these three genes are closely linked) to generate a maintainer line. Progeny produced from self-pollination of the maintainer would segregate half male sterile seeds and half colored maintainer seeds. These two types of seeds can be separated using a color-sorting machine. Cross-pollination of the male sterile line with the pollen lethality maintainer would generate 100% pure male sterile line.

Illuminated by these propositions, scientists at Dupont-Pioneer first developed the seed production technology (referred as SPT in maize) to propagate homozygous male-sterile mutant seeds in maize [15], [16]. They utilized the maize *ms45/ms45* male sterile mutant, transformed it with a construct containing three tightly-linked genes, *i.e.*, the male fertility gene *Ms45*, a maize gene encoding α-amylase (*Zm-aa1*), and the gene encoding red fluorescence protein gene from *Discosoma* sp*.*, *DsRed2*(*Alt1*), to generate a maintainer line. They demonstrated that the *Ms45* male fertility gene can successfully restore male fertility of the *ms45/ms45* mutant, and that the maize α-amylase gene can successfully disrupt pollination process for pollens carrying the transgene, by preventing starch accumulation during pollen maturation and thus depriving energy source required for achieving fertilization. Thus, only the non-transgenic pollens can successfully fertilize the female gametes (transgenic or non-transgenic) to produce progeny. The *DsRed2*(*Alt1*) gene serves as a screenable color marker for identifying the progeny carrying the transgene. According to the classic Mendelian’s law, the progeny produced from selfed maintainer line that carries a single-copy of transgene should segregate non-transgenic kernels (male sterile, normal yellow color) and transgenic kernels (fertile, red fluorescent) in a 1:1 ratio. High-speed optical color sorting was developed to effectively separate these two types of seeds. This technology allows propagation of the recessive nuclear male sterile maize lines, which can be used in commercial breeding program without detasseling, thus helping to reduce the damage associated with detasseling and increase the yield and purity of hybrid seeds. Virtually any fertile maize lines can be used as the male parents to produce F1 hybrids. For propagation of the male sterile line, the maintainer line is used as the male parent to cross with the male sterile mutant (as the female parent), 100% of the progeny will be identical to the original male sterile mutant (non-transgenic). Thus, using the male sterile mutant to produce commercial hybrids does not involve transgene and the obtained hybrid seeds are also non-transgenic. This technology has been deployed in the United States for hybrid maize production since 2012, and the produced hybrids are acknowledged as non-transgenic by regulatory agencies in the United States, Australia, and Japan [16].

It should be noted that although the aforementioned “three-linked-genes” approach offers several benefits in hybrid maize breeding, it should be particularly useful for crops with bisexual flowers and thus not amenable to mechanical or manual emasculation, such as rice and wheat. In fact, this new approach could fundamentally change the hybrid rice breeding strategy [17]. Inspired by this new approach’s success in maize and the intrinsic problems associated with the first-generation (three-line) and second-generation (two-line) hybrid rice systems, a group of scientists including both authors gathered support from various government sources in China, including the Ministry of Science and Technology, Hunan Province Government, Guangdong Province Government, and Shenzhen Municipal Government, to develop the “third-generation” hybrid rice in China. For proof-of-concept study, an approach as shown in Figure 1 was followed. They initially used the *ms26/ms26* male sterile rice mutant (in a *japonica* background), and transformed it with various constructs of different orientations and orders for the three closely-linked genes, *i.e.*, the wild type male fertile gene *Ms26*, *Zm-aa1*, and the gene encoding DsRed2 (with optimized codon usage in rice). The rice *Ms26* gene (OsCYP704B2) encodes a cytochrome P450 monooxygenase responsible for catalyzing the ω-hydroxylation of C16 and C18 fatty acid in tapetum [18]. A large number of transformants (maintainer lines) were generated and characterized molecularly and phenotypically. The best lines (showing complete male fertility restoration, single copy transgene, 1:1 ratio segregation of viable and non-viable pollen grains, and 1:1 segregation ratio of normal colored seeds and pink-colored seeds) were selected for environmental safety and food safety evaluations starting in the spring of 2010. It had been shown that these maintainer lines were as safe as the conventional rice cultivars. This initial work was summarized in a prior report [17].

**Figure 1:**
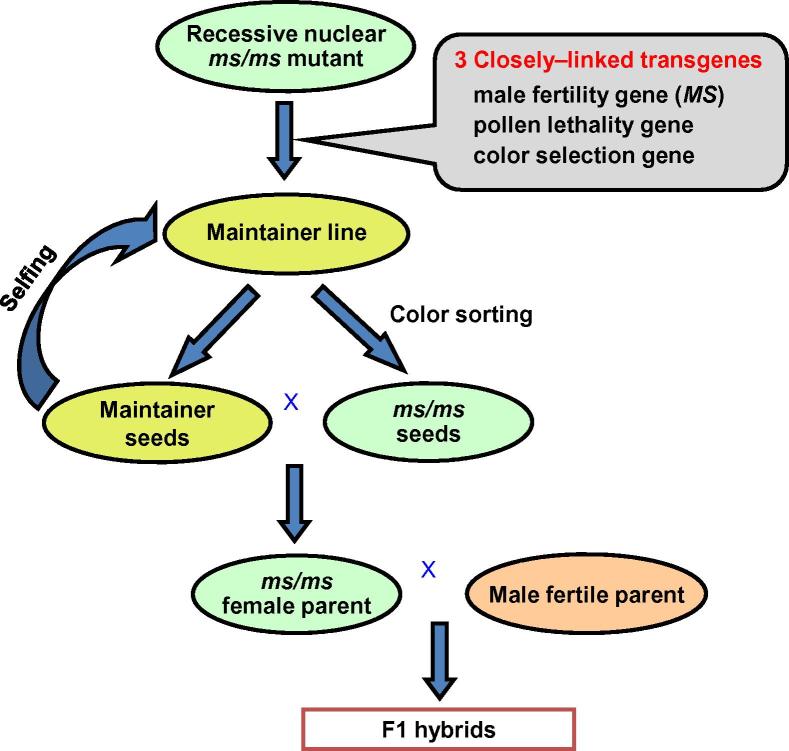
**Diagram scheme of the “third-generation” hybrid rice technology**

Following these efforts, they switched to development and implementation of the “third-generation” hybrid rice system in *indica* rice for the commercialization purpose [19]. They identified a new recessive nuclear male sterile rice mutant, *Oryza sativa No Pollen 1 (osnp1*) from an ethyl methanesulfonate (EMS)-mutagenized population of Huanghuazhan (HHZ), a popular *indica* cultivar of superior grain quality and is widely cultivated in China. Positional cloning revealed that *OsNP1* encodes a glucose-methanol-choline-oxidoreductase required for tapetum degeneration and pollen exine formation [19]. They constructed a binary vector containing two separate T-DNAs; one has the *NPTII* gene under CaMV 35S promoter (serving as a selection marker during the transformation process), and the other contains three gene cassettes: *OsNP1* gene driven by its native promoter, *Zm-aa1* driven by the pollen-specific *PG47* promoter, and *DsRed* driven by the aleurone layer-specific *LTP2* promoter. Both T-DNAs were transformed into the *osnp1* mutant. After screening and characterization of thousands of transformants, one T1 plant (named Zhen18B), with a single-copy transgene of the second T-DNA but lacking the first T-DNA, was selected as the maintainer line. This plant exhibited normal vegetative and reproductive development, and selfing of this plant produces seeds that segregate in 1:1 ratio of fluorescent seeds (with the transgene) and non-fluorescent seeds (no transgene) (Figure 2). The plants from the red fluorescent seeds are identical to the Zhen18B maintainer itself, while all plants from the non-fluorescent seeds (named as Zhen18A) are identical to the *osnp1* mutant. Thus, Zhen18B can be maintained through self-pollination and selection of red fluorescent seeds via seed sorting and Zhen18A can be used as the male sterile line (female parent) for commercial hybrid seed production. As a test, Zhen18A was used as the female parent and cross-pollinated with ∼1200 paternal lines. Approximately 85% of the F1 offsprings had higher yields (on per-plant yield scale) than their parents, and 10% surpassed the best-yielding local varieties. These results demonstrated the promising utility of such a system for hybrid rice breeding in the future. Indeed, Zhen18A was recently approved by the Crop Variety Appraisal Committee of the Guangdong Province. Moreover, a number of hybrids developed using this male sterile lines are in the pipeline of variety registration approval by the Chinese government.

**Figure 2:**
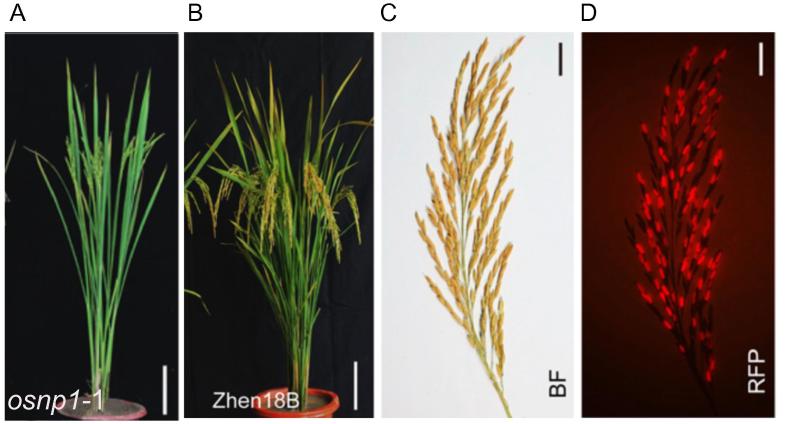
**Development of Zhen18B maintainer line and Zhen18A male sterile line A.** Phenotype of the *osnp1* mutant after bolting. **B.** Phenotype of Zhen18B maintainer plant after bolting. **C.** Panicle of Zhen18B maintainer plant under bright field (BF). **D.** Panicle of Zhen18B maintainer plant under a red fluorescence filter (RFP). The image was adopted from [19].

It should be emphasized that the “third-generation” hybrid system described here has several important advantageous over the “first-generation” and “second-generation” hybrid rice systems. First, virtually any rice germplasms with the wild type fertility gene can restore the fertility of the male sterile mutant caused by a recessive nuclear mutation, thus, greatly expanding the choices of germplasms of paternal lines and increasing the likelihood of breeding superior hybrids with strong heterosis. Second, as the male sterility of the female parent is stable and not influenced by climate or environmental changes, risks associated with unpredictable changes in weather conditions for both propagation of the male sterile seeds and production of hybrid seeds can be eliminated. Third, the male sterility locus and the transgene can be readily introgressed into other backgrounds to generate new pairs of maintainer and male sterile lines through traditional molecular marker-assisted breeding. Finally, transgenic oversight is only applicable to the propagation step of the maintainer line and sterile female line seeds, which requires relatively a small area of fenced land certified for transgenic rice planting, while commercial production and cultivation of hybrid seeds do not require transgenic oversight, as these processes do not involve transgenic plants. Indeed, we believe that the “third-generation” hybrid rice technology (and its utilization in other crops) should hold great promise to further boost hybrid rice (and other crops) production and contribute to solving the daunting task of feeding the world with an ever-increasing population and anticipated adverse climate changes [20].

## Competing interests

The authors declare no competing interests.
